
JOURNAL INFORMATION
==============================
NLM Title Abbreviation: ScientificWorldJournal
Journal ID: ScientificWorldJournal
NLM Journal ID: 101131163
Journal ID: TSWJ

The Scientific World Journal

ISSN: 2356-6140
EISSN: 1537-744X
Publisher: Wiley

ARTICLE INFORMATION
==============================
PMCID: PMC5901231
PMID: 17982580
DOI: 10.1100/tsw.2007.248
Article ID: 924313
Article version: 1
Subjects: Editorial

Glial Cells and the Neurobiology of Addiction

Lawrence Diane M. P. *
Thomas David A.
Wu Da-Yu
Division of Basic Neurosciences and Behavioral Research, National Institute on Drug Abuse, 6001 Executive Boulevard, Rm 4282, MSC 9555, Bethesda, MD 20892-9555, USA
*Diane M. P. Lawrence: lawrencedi@nida.nih.govAcademic Editors: D. Shurtleff and S. Ferre

Publication date: 2007
Electronic publication date: 2007 Nov 2
Volume: 7
First page: 86
Last page: 88
Received 2007 Aug 28; Accepted 2007 Aug 28
Copyright: Copyright © 2007 Diane M. P. Lawrence et al.
Copyright year: 2007
License: This is an open access article distributed under the Creative Commons Attribution License, which permits unrestricted use, distribution, and reproduction in any medium, provided the original work is properly cited.
License URL: https://creativecommons.org/licenses/by/3.0/

==============================
Editorial
NIDA Special Issue on Frontiers in Addiction Research
*TheScientificWorldJOURNAL* (2007) 7(S2), 86–88
ISSN 1537-744X; DOI 10.1100/tsw.2007.248

TheScientificWorldJOURNAL
www.thescientificworld.com

### Glial Cells and the Neurobiology of Addiction

Diane M.P. Lawrence*, David A. Thomas, and Da-Yu Wu

*Division of Basic Neurosciences & Behavioral Research, National Institute on Drug Abuse, 6001 Executive Boulevard, Rm 4282, MSC 9555, Bethesda, MD 20892-9555*

**E-mail:** LawrenceDi@nida.nih.gov

*Received August 28, 2007; Accepted August 28, 2007; Published November 2, 2007*

---

Within the central nervous system, glial cells play significant roles in guiding neural development, synaptogenesis, homeostatic regulation of neuronal function, and neuroplasticity, as well as mediating immune surveillance and inflammatory responses. Activated astrocytes and microglia release a host of cytokines, chemokines, complement, and growth factors that modulate brain development, neuronal function, and inflammation through multiple signaling pathways throughout the life span. These products can be beneficial, guiding neuronal migration, regulating synapse formation, modulating synaptic strength, and providing neuroprotection against cellular damage. However, these factors can also be detrimental, leading to neurotoxicity and neurodegeneration. Recent studies suggest that glial functions are altered by substance abuse, yet much is unknown about the interaction of drugs of abuse and their receptors with glia and neuroinflammatory processes within the CNS.

Some research has begun to elucidate the mechanisms of glial regulation of synapse formation, synapse maintenance, pre- and postsynaptic function, and neuroplasticity. Astrocyte-derived thrombospondin was shown to mediate promotion of CNS synaptogenesis[1,2]. Once synapses are formed, astrocytes also appear to enhance synaptic stability[3], suggesting their importance throughout and beyond development. Besides promoting functional synaptic formation, astrocytes also have a role in synaptic elimination. Recent information suggests that complement factors from astrocytes mediate both protective and pathologic functions[4], as well as initiate developmental synaptic elimination and segregation in CNS (see this issue). These findings point out the active role of glial cells in “sculpting” the brain during development and beyond.

Presynaptic function can be affected by glial-derived factors, such as cholesterol, which was shown to increase evoked synaptic responses most likely by increasing both the number of synaptic vesicles and the formation of vesicle release machinery[1,5]. Glia have been shown to regulate postsynaptic glutamatergic signaling; in some cases this phenomenon occurs *via* TNF-alpha and other molecules increasing postsynaptic glutamate receptor levels[6]. This finding highlights the important role played by glia after injury, when microglia become active producers of TNF-alpha. *In vitro* studies have also suggested that astrocytes affect GABAergic transmission, which in turn modulates neuroplasticity. New information suggests that glia are capable of pruning neuronal dendrites and releasing neurotransmitters, demonstrating further potential mechanisms of glial contributions to synapse elimination and neuroplasticity[7].

Glial activation and neuroinflammation are involved in the etiology of a variety of neurological disorders, such as stroke, spinal cord injury, multiple sclerosis, Alzheimer’s disease, ALS, Parkinson’s disease, chronic inflammatory pain, and AIDS-associated dementia. Immunological, physiological, and psychological stressors engage cytokines and other immune molecules to mediate interactions with neuroendocrine, neuropeptide, and neurotransmitter systems. Brain cytokine levels increase following

*Corresponding author.
©2007 with author.
Published by TheScientificWorld; www.thescientificworld.com exposure to stress, while treatments designed to alleviate stress reverse this effect. However, cytokines and chemokines also modulate CNS function in the absence of overt challenges. For example, inhibitors of cytokines and cytokine receptors affect cognitive and emotional processes, and genetic manipulation to eliminate inflammatory molecules such as TNF-alpha results in neurological impairment, suggesting that inflammatory molecules expressed in the brain are functional under nonpathologic conditions. General strategies to limit neurodegeneration by suppressing glial activation and inflammation are not likely to be completely effective, as this would also inhibit the ability of these same cells to promote CNS repair and regeneration[8].

Neurotransmitter transporters (e.g., glutamate, dopamine) and receptors for drugs of abuse are also localized on astrocytes and microglial cells, so it is expected that substance abuse may disrupt glial function in ways that subsequently alter neuronal function and communication. Recent evidence suggests that methamphetamine-induced damage to dopamine neuron terminals is associated with microglial activation[9], and may be mediated by the cytokine TNF-alpha, oxidative stress, and/or matrix metalloproteinases. Cocaine can affect astrocyte and microglial function and protein expression[10,11], but the receptor and signaling pathways involved are unclear. Cannabinoid receptors are expressed by astrocytes and microglia, and their activation can inhibit glutamate uptake by astrocytes, reduce microglial activation and inflammatory responses, and protect against memory impairment and microglial-mediated neuronal damage[12,13]. Functional opioid receptors have been found on astrocytes and their activation may be involved in the development of opioid analgesic tolerance, but the mechanisms for this phenomenon are only now being explored[14].

A direct role of glial involvement in drug addiction may be highlighted by the recent reports that the astrocyte itself can be excited by glutamate and can also release glutamate in the nucleus accumbens[15,16], the center of reward and reinforcement processing in the brain. How the abnormal fine tuning of astrocytes during substance abuse reinforces the drug effect and leads to addiction will be one of the most challenging questions in drug abuse research.

Clearly, this is a time of great advances in our understanding of the role glial cells play in brain function and the impact of drugs of abuse in particular, however, much is still to be discovered. Issues for future research include understanding the functional interactions among abused substances and their endogenous counterparts with astrocytes and microglia, neurons, and inflammatory molecules that mediate glial-neuronal communication. Research is needed to determine how drugs of abuse affect the homeostatic feedback control of neuronal function provided by glial cells, how substance abuse changes the neuroprotective and neuropathogenic functions of glia, and how substance abuse affects neuronal communication with glia. The functional roles of inflammatory factors including cytokines, chemokines, and complement in synaptic or dendritic pruning also require study. The interactions between glial- and neuron-derived neurotrophic factors, such as glial-derived neurotrophic factor (GDNF), brain-derived neurotrophic factor (BDNF), and conserved dopamine neurotrophic factor (CDNF) in neuronal plasticity, and the means by which abused substances interfere with their regulation, will be another area critical for progress in neurobiology. Finally, interdisciplinary research addressing glial-neuronal communication as it affects neural circuit development, neuroplasticity, neurotoxicity, and pain, as well as the mechanisms of substance abuse interactions with these processes, are extremely important and will have a significant impact on our understanding of substance abuse and addiction processes in the brain, as well as on fundamental mechanisms of neurobiology.

### REFERENCES

1. Ullian, E.M., Christopherson, K.S., and Barres, B.A. (2004) Role for glia in synaptogenesis. *Glia* **47,** 209–216.
2. Christopherson, K.S., Ullian, E.M., Stokes, C.C., Mullowney, C.E., Hell, J.W., Agah, A., Lawler, J., Mosher, D.F., Bornstein, P., and Barres, B.A. (2005) Thrombospondins are astrocyte-secreted proteins that promote CNS synaptogenesis. *Cell* **120,** 421–433.
3. Ullian, E.M., Sapperstein, S.K., Christopherson, K.S., and Barres, B.A. (2001) Control of synapse number by glia. *Science* **291,** 657–661.

4. Griffiths, M., Neal, J.W., and Gasque, P. (2007) Innate immunity and protective neuroinflammation: new emphasis on the role of neuroimmune regulatory proteins. *Int. Rev. Neurobiol.* **82,** 29–55.
5. Mauch, D.H., Nagler, K., Schumacher, S., Goritz, C., Muller, E.C., Otto, A., and Pfrieger, F.W. (2001) CNS synaptogenesis promoted by glia-derived cholesterol. *Science* **294,** 1354–1357.
6. Beattie, M.S., Hermann, G.E., Rogers, R.C., and Bresnahan, J.C. (2002) Cell death in models of spinal cord injury. *Prog. Brain Res*. **137,** 37–47.
7. Allen, N.J. and Barres, B.A. (2005) Signaling between glia and neurons: focus on synaptic plasticity. *Curr. Opin. Neurobiol*. **15,** 542–548.
8. Melchior, B., Puntambekar, S.S., and Carson, M.J. (2006) Microglia and the control of autoreactive T cell responses. *Neurochem. Int*. **49,** 145–153.
9. Kuhn, D.M., Francescutti-Verbeem, D.M., and Thomas, D.M. (2006) Dopamine quinones activate microglia and induce a neurotoxic gene expression profile: relationship to methamphetamine-induced nerve ending damage. *Ann. N. Y. Acad. Sci.* **1074,** 31–41.
10. Reynolds, J.L., Mahajan, S.D., Bindukumar, B., Sykes, D., Schwartz, S.A., and Nair, M.P. (2006) Proteomic analysis of the effects of cocaine on the enhancement of HIV-1 replication in normal human astrocytes (NHA). *Brain Res*. **1123,** 226–236.
11. Griffin, W.C., 3rd, Middaugh, L.D., and Tyor, W.R. (2007) Chronic cocaine exposure in the SCID mouse model of HIV encephalitis. *Brain Res*. **1134**, 214–219.
12. Eljaschewitsch, E., Witting, A., Mawrin, C., Lee, T., Schmidt, P.M., Wolf, S., Hoertnagl, H., Raine, C.S., Schneider-Stock, R., Nitsch, R., and Ullrich, O. (2006) The endocannabinoid anandamide protects neurons during CNS inflammation by induction of MKP-1 in microglial cells. *Neuron* **49,** 67–79.
13. Witting, A., Chen, L., Cudaback, E., Straiker, A., Walter, L., Rickman, B., Moller, T., Brosnan, C., and Stella, N. (2006) Experimental autoimmune encephalomyelitis disrupts endocannabinoid-mediated neuroprotection. *Proc. Natl. Acad. Sci. U. S. A.* **103,** 6362–6367.
14. Watkins, L.R., Hutchinson, M.R., Johnston, I.N., and Maier, S.F. (2005) Glia: novel counter-regulators of opioid analgesia. *Trends Neurosci*. **28,** 661–669.
15. D'Ascenzo, M., Fellin, T., Terunuma, M., Revilla-Sanchez, R., Meaney, D.F., Auberson, Y.P., Moss, S.J., and Haydon, P.G. (2007) mGluR5 stimulates gliotransmission in the nucleus accumbens. *Proc. Natl. Acad. Sci. U. S. A.* **104,** 1995–2000.
16. Lee, S.Y. and Haydon, P.G. (2007) Astrocytic glutamate targets NMDA receptors. *J. Physiol*. **581**, 887–888.

**This article should be cited as follows:**

Lawrence, D.M.P., Thomas, D., and Wu, D.-Y. (2007) Glial cells and the neurobiology of addiction. *TheScientificWorldJOURNAL* **7(S2)**, 86–88. DOI 10.1100/tsw.2007.248.
